# Current status of newborn screening for Pompe disease in Japan

**DOI:** 10.1186/s13023-021-02146-z

**Published:** 2021-12-18

**Authors:** Takaaki Sawada, Jun Kido, Keishin Sugawara, Ken Momosaki, Shinichiro Yoshida, Kanako Kojima-Ishii, Takahito Inoue, Shirou Matsumoto, Fumio Endo, Shouichi Ohga, Shinichi Hirose, Kimitoshi Nakamura

**Affiliations:** 1 Department of Pediatrics, Graduate School of Medical Sciences, Kumamoto University, 1-1-1 Honjo, Chuo-ku, Kumamoto City, Kumamoto 860-8556 Japan; 2 Kumamoto-Ashikita Medical Center for Disabled Children, Kumamoto, Japan; 3 KM Biologics Co., Ltd., Kumamoto, Japan; 4 Department of Pediatrics, Graduate School of Medical Sciences, Kyushu University, Fukuoka, Japan; 5 Department of Pediatrics, School of Medicine, Fukuoka University, Fukuoka, Japan; 6 Department of Pediatrics, Fukuoka University Chikushi Hospital, Fukuoka, Japan; 7 Kumamoto-Ezuko Medical Center for Disabled Children, Kumamoto, Japan; 8 General Medical Research Center, School of Medicine, Fukuoka University, Fukuoka, Japan

**Keywords:** Pompe disease, Newborn screening, Acid α-glucosidase, *GAA*, Pseudodeficiency allele, Disease frequency

## Abstract

**Background:**

Pompe disease is an autosomal recessive inherited metabolic disorder caused by a deficiency of the acid α-glucosidase (GAA). Pompe disease manifests as an accumulation of lysosomal glycogen in the skeletal and heart muscle. We conducted newborn screening (NBS) for Pompe disease in Japan from April 2013 to October 2020 to determine the feasibility and utility of NBS for Pompe disease.

**Results:**

From the 296,759 newborns whose enzyme activity was measured, 107 of which underwent *GAA* analysis, we found one patient with infantile-onset Pompe disease (IOPD) and seven with potential late-onset Pompe disease (LOPD). We identified 34 pseudodeficient individuals and 65 carriers or potential carriers. The frequency of patients with IOPD was similar to that in the United States, but significantly lower than that in Taiwan. One patient with IOPD underwent early enzyme replacement therapy within a month after birth before presenting exacerbated manifestations, whereas those with potential LOPD showed no manifestations during the follow-up period of six years.

**Conclusions:**

The frequency of IOPD in Japan was similar to that in the United States, where NBS for Pompe disease is recommended. This indicates that NBS for Pompe disease may also be useful in Japan. Therefore, it should be used over a wider region in Japan.

**Supplementary Information:**

The online version contains supplementary material available at 10.1186/s13023-021-02146-z.

## Background

Pompe disease, also known as glycogen storage disease type II (OMIM 232300), is an autosomal recessive inherited metabolic disorder caused by a deficiency of the enzyme known as acid α-glucosidase (GAA, EC 3.2.1.20/3) that breaks down glycogen in the lysosome, leading to the accumulation of lysosomal glycogen in skeletal and heart muscles [1]. In addition, glycogen accumulates in tissues throughout the body and may cause symptoms in multiple organs, including the nervous system [2]. Clinically, two classical phenotypes have been described according to the age of onset: infantile and late-onset types [3]. Patients with infantile-onset Pompe disease (IOPD) exhibit a nearly complete absence of GAA activity and present with hypotonia and hypertrophic cardiomyopathy in early infancy. These patients eventually die of cardiorespiratory failure within the first year of life because of the extensive accumulation of glycogen in skeletal and heart muscles. In contrast, patients with late-onset Pompe disease (LOPD) have a variable residual GAA activity and predominantly manifest skeletal muscle dysfunction but rarely present cardiac muscle dysfunction. Enzyme replacement therapy (ERT) is essential for the treatment of IOPD [4, 5]. To achieve optimal outcomes, ERT should be started before symptoms clearly manifest and prior to the development of irreversible damage [6, 7]. The early initiation of ERT in patients with IOPD can improve survival rates and quality of life, reduce the need for ventilation, and promote earlier independent walking [8, 9].

Newborn screening (NBS) is one of the best approaches for the early diagnosis and treatment of Pompe disease. Asian populations have a high frequency of pseudodeficiency alleles c.1726G > A and 2065G > A in *GAA*, which significantly reduce GAA activity [10]. Particularly, in the Japanese population, the frequency of these alleles is estimated to be 3.9% and 30.5% in homozygous and heterozygous forms, respectively [11]. Therefore, the presence of pseudodeficiency alleles are major obstacles that have negatively affected NBS for Pompe disease in our previous pilot program, which analyzed 715 Japanese newborns and 18 previously diagnosed patients [11]. Some NBS programs for Pompe disease, including ours, have shown that the combination of GAA enzyme assays using dried-blood spot (DBS) cards and *GAA* gene mutation analysis could be useful in distinguishing false-positive cases from patients with Pompe disease.

Previously, we conducted a screening program for Pompe disease among 103,204 newborns in the Kumamoto and Fukuoka prefectures in Japan, and we identified no patient with IOPD and only three individuals with potential LOPD [12]. Therefore, in this study, we conducted the program at a larger scale, screening 297,387 newborns from April 2013 to October 2020 by employing a fluorometric enzymatic assay with 4-methylumbelliferyl α-D-glucopyranoside (4MU-αGlc) on DBSs to detect GAA activity. We identified a patient with IOPD and detected individuals with potential LOPD. This is the first report of the determination of the frequency of IOPD in Japan through NBS.

## Results

### NBS for Pompe disease

**Fig. 1:**
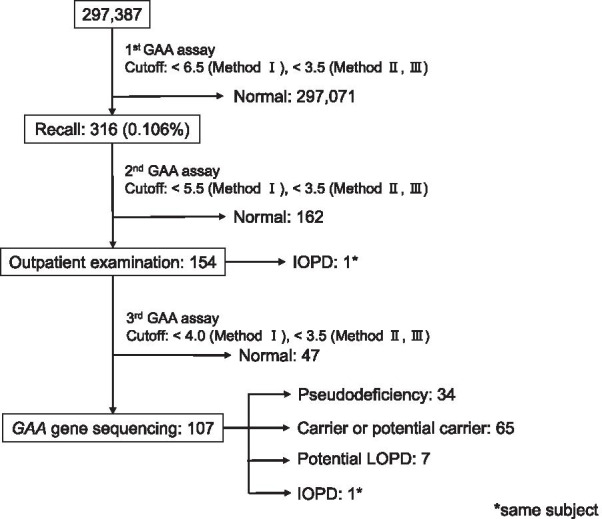
Flowchart of the newborn screening program for Pompe disease

**Fig. 2:**
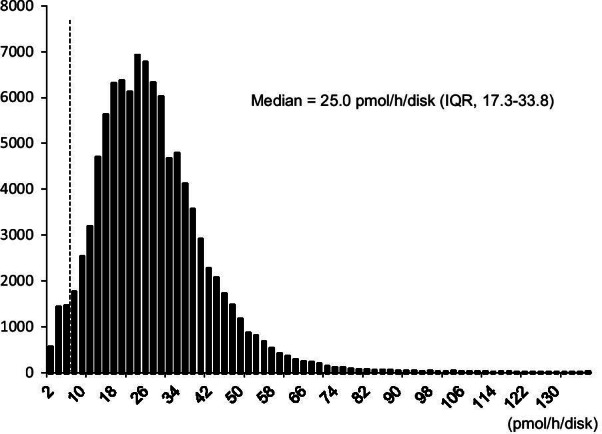
Histograms of GAA activity in the newborns. Histograms of GAA activity are shown for Method I (*N* = 99,936) in the newborns. Dashed line indicates the cutoff level

The experimental workflow and the results of our NBS program for Pompe disease are shown in Fig. 1. In total, 297,387 newborns were screened. We established efficient GAA assay methods I to III by modifying the reaction time and buffer composition to achieve multiple high-throughput screening options. In summary, the median GAA activities were 25.0 (interquartile range (IQR), 17.3–33.8) (Fig. 2), 45.4 (31.8–62.5), and 40.0 pmol h^−1^ disk^−1^ (28.6–53.1) in Methods I–III, respectively (Additional file 1). Next, 316 DBSs whose GAA activities were under the cutoff values (< 6.5 pmol h^−1^ disk^−1^ in Method I; < 3.5 pmol h^−1^ disk^−1^ in Methods II, III) were recalled for a second GAA assay. The cutoff value for Method I was set at the 4th percentile in the pilot study, whereas those for Methods II and III were set at the 0.1 percentile. Moreover, 154 newborns whose GAA activities were under the cutoff for the second GAA assay were examined at the outpatient clinic. Physical and biochemical examinations, including that for alanine transaminase, aspartate aminotransferase, lactate dehydrogenase, and creatine kinase (CK), and echocardiogram assessments were performed at referral hospitals. The third GAA assay was then performed. The DBSs of 107 newborns whose GAA activities were under the cutoff for the third GAA assay were subjected to *GAA* gene sequencing analysis. Overall, we identified one patient with IOPD and seven individuals as potential LOPD patients.

### Detected variants

The *GAA* gene is highly polymorphic, and many novel variants are continually being discovered. Based on the Pompe disease *GAA* variant database [13] (http://www.pompevariantdatabase.nl; Accessed March 3, 2021) and the ClinVar Miner database [14] (https://clinvarminer.genetics.utah.edu/; Accessed March 3, 2021), we identified 910 and 1,170 sequence variations, respectively. Table 1 shows the 78 variants identified from 107 newborns, 71 of which were registered in the Pompe disease *GAA* variant database [13, 15] or ClinVar Miner. The other seven variants, i.e., c.439G > A (p.G147S), c.539_543delACTTC (p.F181Dfs*6), c.1170delC (p.N390Kfs*2), c.547-27A > G, c.1194 + 19_1194 + 20 insA, c.1552-52C > A, and c.2859G > A (non-coding), were not registered. The last two variants are considered as nonpathogenic according to our previous report [12], whereas the other five are novel. One missense variant, c.439G > A (p.G147S), was predicted by using PolyPhen-2 as “probably damaging.” Two variants, c.539_543delACTTC (p.F181Dfs*6) and c.1170delC (p.N390Kfs*2) had frameshift changes and were predicted as pathogenic. The pathogenicity of two intronic variants, c.547-27A > G and c.1194 + 19_1194 + 20 insA, are unknown; however, c.547-27A > G was predicted using the Human Splicing Finder [16] as a “potential alteration of splicing.” Therefore, we focused on four variants predicted as disease-associated, namely c.439G > A (p.G147S), c.539_543delACTTC (p.F181Dfs*6), c.1170delC (p.N390Kfs*2), and c.547-27A > G.

**Table 1:** Variants detected in the NBS program for Pompe disease

	Variant No	rs IDs	Nucleic acid	Amino acid	Location	Classification		
						Pompe disease *GAA* variant database	ClinVar Miner	PolyPhen-2 (score)
Disease-associated variants	2	rs772534106	c.317G > A	p.R106H	Exon 2	Potentially less severe	Uncertain significance	Probably damaging (1.000)
	4	–	c.439G > A	p.G147S	Exon 2	NR	NR	Probably damaging (0.989)
	5	rs376685205	c.503G > C	p.R168P	Exon 2	Unknown	Uncertain significance	Possibly damaging (0.795)
	6	–	c.539_543delACTTC	p.F181Dfs*6	Exon 2	NR	NR	–
	7	rs143523371	c.546G > T	p.T182 =	Exon 2	Potentially mild	Likely pathogenic	–
	8	rs756024023	c.546 + 5G > T	–	Intron 2	Unknown	Conflicting interpretations of pathogenicity	–
	14	–	c.547-27A > G	–	Intron 2	NR	NR	##
	21	rs200856561	c.752C > T	p.S251L	Exon 4	Presumably non-pathogenic	Conflicting interpretations of pathogenicity	Benign (0.004)
	22	rs577915581	c.761C > T	p.S254L	Exon 4		Pathogenic/Likely pathogenic	Probably damaging (0.997)
	23	rs1555599667	c.796C > T	p.P266S	Exon 4	Potentially mild	Likely pathogenic	Benign (0.001)
	28	–	c.1082C > A	p.P361Q	Exon 7	Potentially less severe	NR	Probably damaging (1.000)
	29	rs142752477	c.1124G > A	p.R375H	Exon 7	Unknown	Uncertain significance	Probably Damaging (1.000)
	31	–	c.1170delC	p.N390Kfs*2	Exon 7	NR	NR	–
	32	rs776008078	c.1190C > T	p.P397L	Exon 7	Less severe	Uncertain significance	Probably damaging (1.000)
	35	–	c.1244C > T	p.T415M	Exon 8	Potentially less severe	NR	Probably damaging (1.000)
	36	rs770610356	c.1309C > T	p.R437C	Exon 8	Less severe	Pathogenic/likely pathogenic	Probably damaging (0.995)
	37	rs747610090	c.1316 T > A	p.M439K	Exon 8	Potentially mild	Pathogenic/likely pathogenic	Benign (0.096)
	43	–	c.1494G > A	p.W498*	Exon 10	Very severe	NR	–
	46	–	c.1562A > T	p.E521V	Exon 11	Unknown	Uncertain significance	Probably damaging (1.000)
	50	rs747150965	c.1669A > T	p.I557F	Exon 12	Very severe	Conflicting interpretations of pathogenicity	Benign (0.069)
	52	rs764670084	c.1798C > T	p.R600C	Exon 13	Less severe	Pathogenic	probably damaging (1.000)
	53	rs914396317	c.1857C > G	p.S619R	Exon 13	Less severe	Likely pathogenic	Probably damaging (1.000)
	55	rs28940868	c.1935C > A	p.D645E	Exon 14	Potentially less severe	Pathogenic	Probably damaging (1.000)
	56	rs1555601633	c.2003A > G	p.Y668C	Exon 14	Potentially less severe	Uncertain significance	Probably damaging (1.000)
	58	–	c.2055C > G	p.Y685*	Exon 15	Very severe	NR	–
	62	rs121907938	c.2173C > T	p.R725W	Exon 15	Less severe	Pathogenic	Probably damaging (1.000)
	64	rs1800312	c.2238G > C	p.W746C	Exon 16	Potentially mild	Pathogenic/likely pathogenic	Probably damaging (1.000)
	71	rs121907943	c.2560C > T	p.R854*	Exon 18	Very severe	Pathogenic	–
	72	rs192679574	c.2647-7G > A	–	Intron 18	Potentially mild	Pathogenic/likely pathogenic	–
non-pathogenic variants	1	–	c.-260G > C	–	Exon 1	Non-pathogenic	Likely benign	–
	3	–	c.324 T > C	p.C108 =	Exon 2	Non-pathogenic	Benign	–
	9	rs34746710	c.546 + 293G > A	–	Intron 2	Non-pathogenic	Benign	–
	10	rs8065426	c.547-243C > G	–	Intron 2	Non-pathogenic	Benign	–
	11	rs12452263	c.547-238 T > C	–	Intron 2	Non-pathogenic	Benign	–
	12	–	c.547-67C > G	–	Intron 2	Non-pathogenic	Benign	–
	13	rs12452721	c.547-39 T > G	–	Intron 2	Non-pathogenic	Benign	–
	15	rs3816256	c.547-4C > G	–	Intron 2	non-pathogenic	benign	–
	16	rs1042393	c.596A > G	p.H199R	Exon 3	Non-pathogenic	Benign	0.000 (benign)
	17	rs1042395	c.668G > A	p.R223H	Exon 3	Non-pathogenic	Benign	0.206 (benign)
	18	–	c.692 + 38C > T	–	Intron 3	Non-pathogenic	NR	–
	19	rs11150844	c.693-216 T > C	–	Intron 3	Non-pathogenic	Benign	–
	20	rs2304846	c.705G > A	p.T235 =	Exon 4	Unknown	Conflicting interpretations of pathogenicity	–
	24	–	c.858 + 5_858 + 6ins7GCAGCGG	–	Intron 4	Unknown	NR	–
	25	rs5822325	c.858 + 8G > A	–	Intron 4	NR	Benign/likely benign	–
	26	rs2304845	c.858 + 30 T > C	–	Intron 4	Non-pathogenic	Benign	–
	27	rs2252455	c.955 + 12G > A	–	Intron 5	Non-pathogenic	Benign	–
	30	rs111832449	c.1143C > G	p.T381 =	Exon 7	NR	Conflicting interpretations of pathogenicity	–
	33	–	c.1194 + 19_1194 + 20insA	–	Intron 7	NR	NR	–
	34	rs1800304	c.1203G > A	p.Q401 =	Exon 8	Non-pathogenic	Benign	–
	38	rs6565641	c.1327-269A > G	–	Intron 8	Non-pathogenic	Benign	–
	39	rs2278620	c.1327-179G > A	–	Intron 8	Non-pathogenic	Benign	–
	40	rs2278619	c.1327-18A > G	–	Intron 8	Non-pathogenic	Benign	–
	41	rs2278618	c.1438-220A > G	–	Intron 9	Non-pathogenic	Benign	–
	42	rs2304844	c.1438-19G > C	–	Intron 9	Non-pathogenic	Benign	–
	44	rs2304843	c.1551 + 49C > A	–	Intron 10	Non-pathogenic	Benign	–
	45	–	c.1552-52C > A	–	Intron 10	NR	NR	#
	47	rs1042396	c.1581G > A	p.R527 =	Exon 11	Non-pathogenic	Benign	–
	48	rs2304842	c.1636 + 43G > T	–	Intron 11	Non-pathogenic	Benign	–
	49	rs79487884	c.1636 + 210G > A	–	Intron 11	Non-pathogenic	Benign	–
	51	rs1800307	c.1726G > A†	p.G576S	Exon 12	Presumably non-pathogenic	Benign	Probably damaging (1.000)
	54	rs2304837	c.1888 + 21G > A	–	Intron 13	Non-pathogenic	Benign	–
	57	rs2304836	c.2040 + 20A > G	–	Intron 14	Non-pathogenic	Benign	–
	59	–	c.2065G > A†	p.E689K	Exon 15	Non-pathogenic	Benign, other	0.092 (benign)
	60	rs1555601773	c.2132C > G	p.T711R	Exon 15	Non-pathogenic	Conflicting interpretations of pathogenicity	0.999
(Probably damaging)								
	61	rs1800310	c.2133A > G	p.T711 =	Exon 15	Non-pathogenic	Benign	–
	63	rs7221604	c.2189 + 263G > A	–	Intron 15	Non-pathogenic	Benign	–
	65	rs2304832	c.2331 + 20G > A	–	Intron 16	Non-pathogenic	Benign	–
	66	rs2304831	c.2331 + 24 T > C	–	Intron 16	Non-pathogenic	Likely benign	–
	67	rs2304830	c.2332-198A > T	–	Intron 16	Non-pathogenic	Benign	
	68	rs1126690	c.2338G > A	p.V780I	Exon 17	Non-pathogenic	Benign	0.000 (benign)
	69	rs1800314	c.2446G > A	p.V816I	Exon 17	Non-pathogenic	Benign	0.008 (benign)
	70	rs1042397	c.2553G > A	p.G851 =	Exon 18	Non-pathogenic	benign	–
	73	rs9890469	c.2800-227C > T	–	Intron 19	Non-pathogenic	benign	–
	74	–	c.2859G > A	p.*953 =	Exon 20	NR	NR	#
	75	rs1800317	c.*3G > A	–	Exon 20	Non-pathogenic	Benign	–
	76	rs865903736	c.*139dup	–	Exon 20	NR	Likely benign	–
	77	rs8132	c.*223C > T	–	Exon 20	Non-pathogenic	Benign	–
	78	rs7567	c.*419G > T	–	Exon 20	NR	Benign	–

NR, not registered

^†^Pseudodeficiency alleles; Human Splicing Finder: ^#^No impact on splicing; ^##^Potential alteration of splicing

Of the 78 variants, 29 were classified as disease-associated (pathogenic or predicted pathogenic variants), and the other 49 were classified as non-pathogenic (polymorphisms or predicted nonpathogenic variants) (Table 1). The most common disease-associated variant was c.752C > T + c.761C > T, accounting for 31 alleles (14.5%, 31/214). Meanwhile, c.752C > T and c.761C > T occurred in *cis* formation [17]; thus, these variants were treated as c.752C > T + c.761C > T. The second most common disease-associated variant was c.317G > A (p.R106H), which accounted for 10 alleles (4.7%, 10/214), whereas the third most common was c.1316 T > A (p.M439K), which accounted for seven alleles (3.3%, 7/214).

#### Identified individuals with GAA variants

We detected one individual as IOPD patient (subject ID 140), seven as potential LOPD patients (subject IDs 84, 87, 93, 127, 130, 131, and 133), 65 carriers or potential carriers, and 34 with pseudodeficiency alleles (Table 2). A total of 102 of 107 individuals with low GAA activity (95.3%) had a pseudodeficiency variant, c.[1726G > A; 2065G > A].

**Table 2:** Distribution of mutations or predictable pathogenic variants in each subject

Variant No	Nucleotide change	Amino acid change	Subject ID																																							
			25	34	39	89	99	66	111	122	137	112	132	119	45	118	141	105	42	72	76	100	28	13	128	69	135	29	31	60	74	125	126	146	144	83	33	51	3	17	64	129
2	c.317G > A	p.R106H	○	○	○	○	○	○	○	○	○																															
4	c.439G > A	p.G147S										○																														
5	c.503G > C	p.R168P											○																													
6	c.538_542delCACTT	p.H180Hfs*7																																								
7	c.546G > T	p.T182 =																	○	○	○	○																				
8	c.546 + 5G > T	–												○																												
14	c.547-27A > G	–																																								
21	c.752C > T	p.S251L																																								
22	c.761C > T	p.S254L																																								
23	c.796C > T	p.P266S													○																											
28	c.1082 C > A	p.P361Q																					○																			
29	c.1124 G > A	p.R375H																						○																		
31	c.1170delC	p.N390Kfs*2																							○																	
32	c.1190C > T	p.P397L																								○																
35	c.1244C > T	p.T415M																																								
36	c.1309C > T	p.R437C																									○															
37	c.1316 T > A	p.M439K																										○	○	○	○	○	○	○								
43	c.1494G > A	p.W498*																																	○							
46	c.1562A > T	p.E521V																																		○						
50	c.1669A > T	p.I557F																																								
51	c.1726G > A†	p.G576S	●	●	●	●	●	●	●	●	●	●	●	●	●	●	●	●	○	○	○	○	○	○	○	○	○	○	○	○	○	○	○	○	○	○	○	○	○	○	○	○
52	c.1798C > T	p.R600C														○																										
53	c.1857C > G	p.S619R																																			○					
55	c.1935C > A	p.D645E															○																									
56	c.2003A > G	p.Y668C																																								
58	c.2055C > G	p.Y685*																																								
59	c.2065G > A†	p.E689K	●	●	●	●	●	●	●	●	●	●	●	●	●	●	●	●	○	○	○	○	○	○	○	○	○	○	○	○	○	○	○	○	○	○	○	○	○	○	○	○
62	c.2173C > T	p.R725W																																								
64	c.2238G > C	p.W746C																○																								
71	c.2560C > T	p.R854*																																								
72	c.2647-7G > A	–																																					○	○	○	○
	GAA activity (pmol/h/disk)	DBS	1.8	< 1.0	1.7	< 1.0	2.9	< 1.0	2.5	1.3	2.0	< 1.0	3.2	< 1.0	1.7	< 1.0	2.9	< 1.0	< 1.0	< 1.0	1.8	3.6	1.3	< 1.0	2.6	< 1.0	1.4	< 1.0	1.7	1.3	< 1.0	3.9‡	2.4	2.2	3.1	1.3	< 1.0	< 1.0	1.1	1.7	1.3	3.1
		fibroblast	5.4	18.7	6.1	ND	ND	4.9	ND	ND	ND	ND	ND	ND	8.8	ND	ND	ND	4.2	7.8	ND	2.9	9.6	23.4	ND	4.4	ND	12.6	8.3	7.7	8.7	ND	ND	ND	ND	4.7	6.5	6	5.3	27	ND	ND
	Diagnosis		Carrier or potential carrier (DBS GAA range: < 1.0–3.6)																																							

Variant No	Nucleotide change	Amino acid change	Subject ID																																
			5	12	23	24	26	40	56	58	59	63	71	73	79	88	96	101	109	113	124	134	139	142	143	106	8	93	87	84	127	131	133	130	140
2	c.317G > A	p.R106H																										○							
4	c.439G > A	p.G147S																																	
5	c.503G > C	p.R168P																																	
6	c.538_542delCACTT	p.H180Hfs*7																																	○
7	c.546G > T	p.T182 =																																	
8	c.546 + 5G > T	–																																	
14	c.547-27A > G	–																																	○
21	c.752C > T	p.S251L	○	○	○	○	○	○	○	○	○	○	○	○	○	○	○	○	○	○	○	○	○	○	○			○	○	○	○	●	●		
22	c.761C > T	p.S254L																																	
23	c.796C > T	p.P266S																																	
28	c.1082 C > A	p.P361Q																																	
29	c.1124 G > A	p.R375H																																	
31	c.1170delC	p.N390Kfs*2																																	
32	c.1190C > T	p.P397L																																	
35	c.1244C > T	p.T415M																												○					
36	c.1309C > T	p.R437C																																	
37	c.1316 T > A	p.M439K																																	
43	c.1494G > A	p.W498*																																	
46	c.1562A > T	p.E521V																																	
50	c.1669A > T	p.I557F																								○									
51	c.1726G > A†	p.G576S	○	○	○	○	○	○	○	○	○	○	○	○	○	○	○	○	○	○	○	○	○	○	○	○	○	○	○						○
52	c.1798C > T	p.R600C																																	○
53	c.1857C > G	p.S619R																													○				
55	c.1935C > A	p.D645E																																	
56	c.2003A > G	p.Y668C																											○						
58	c.2055C > G	p.Y685*																																	
59	c.2065G > A†	p.E689K	○	○	○	○	○	○	○	○	○	○	○	○	○	○	○	○	○	○	○	○	○	○	○	○	●	○	○						○
62	c.2173C > T	p.R725W																																○	
64	c.2238G > C	p.W746C																																	
71	c.2560C > T	p.R854*																									○								
72	c.2647-7G > A	–																																○	
	GAA activity (pmol/h/disk)	DBS	< 1.0	1.1	< 1.0	< 1.0	2.1	1.3	< 1.0	< 1.0	< 1.0	1.3	< 1.0	< 1.0	1.3	< 1.0	< 1.0	< 1.0	< 1.0	< 1.0	< 1.0	2.5	2.1	2.0	1.7	< 1.0	2.0	< 1.0	< 1.0	< 1.0	< 1.0	3.2	< 1.0	1.9	1.0
		Fibroblast	10	ND	ND	ND	35.1	3.7	3	8.1	7.8	ND	ND	7.6	ND	9.6	ND	ND	ND	ND	ND	ND	ND	ND	ND	14.5	38	ND	5.4	3.0	0.9	ND	ND	ND	ND
	Diagnosis		Carrier or potential carrier (DBS GAA range: < 1.0–3.6)																									Potential LOPD (DBS GAA range: < 1.0–3.2)							IOPD

DBS GAA control range: 6.6– > 205

ND, not done; †, pseudodegiciency alleles; ‡, assayed by the Ba/Zn method

●, Homozygote; ○, Heterozygote; LOPD, late-onset Pompe disease; IOPD, infantile-onset Pompe disease

One patient with IOPD had two pathogenic variants, c.539_543delACTTC (p.F181Dfs*6) and c.1798C > T (p.R600C), and one predicted pathogenic variant, c.547-27A > G. The c.539_543delACTTC variant was novel, whereas c.1798C > T has been reported as pathogenic in patients with IOPD [18]. In addition, this patient developed hypotonia, hypertrophic cardiomyopathy, and hyperCKemia (714 IU/L) within a month after birth, and was subjected to ERT in the first month of life. On day 3 of treatment, the hypotonia improved, and the hypertrophic cardiomyopathy improved after 10 weeks. At 7 months of age, this patient had mild hyperCKemia (200–300 IU/L) but had normal psychomotor development.

Seven individuals who presented with more than two disease-associated variants did not develop any signs or symptoms related to Pompe disease (e.g., hypotonia, echocardiographic findings of hypertrophic cardiomyopathy, and hyperCKemia) and, thus, received no treatment. They were classified as potential LOPD patients. Their GAA activities and gene mutations are shown in Table 2. Six individuals had c.752C > T + c.761C > T. The other variants included c.317G > A (p.R106H), c.1244C > T (p.T415M), c.1857C > G (p.S619R), c.2003A > G (p.Y668C), c.2173C > T (p.R725W), and c.2647-7G > A. Three of these seven individuals (i.e., subject ID 84, 87, and 93) had been previously identified [12] and were subjected to follow-up evaluation. As of February 2021, they remained free from signs or symptoms associated with Pompe disease.

## Discussion

Previously, we have reported the first large-scale newborn screening (*N* = 103,204) for Pompe disease in Japan and identified no IOPD patients and three potential LOPD patients [12]. By October 2020, 297,387 newborns have been screened, and one patient with IOPD and four potential newborns with LOPD were identified. The patient with IOPD was able to undergo early ERT in the first month of life before presenting with exacerbated symptoms.

This screening program demonstrated the effectiveness of NBS for Pompe disease both in Japan and overseas. The frequency of IOPD detected in this program (i.e., 1/296,759) is similar to that in the United States (i.e., California 1/226,000 [19], Illinois 1/228,000 [20], and Pennsylvania 1/265,570 [21]). In contrast, the NBS for Pompe disease in Taiwan identified six patients with IOPD from 402,281 newborns (1/57,000) [9]. Although Japan and Taiwan are geographically near, the rate of detection of IOPD pathogenic variants is different. In Taiwan, five of six IOPD patients are homozygous or heterozygous for the same variant c.1935C > A. In contrast, our study only detected one individual (No. 141) with the same variant among 107 newborns who underwent *GAA* sequencing analysis (allele frequency: 0.47%) (Table 2). The high frequency of IOPD patients in Taiwan is attributed to the high frequency of allele c.1935C > A, which was most commonly found in IOPD patients in Taiwan and southern China [22–24]. On the other hand, the Missouri program [25] detected more patients with IOPD than our program. Particularly, 10 patients with IOPD were identified from approximately 467,000 newborns (1/46,700). In addition, c.525delT was detected in three alleles, whereas c.1447G > A, c.1802C > T, c.2560C > T, c.-32-13T > G, and del exon 18 were detected in two alleles. The high frequency of these specific variants may contribute to the high frequency of patients with IOPD.

The IOPD patient identified in this study harbored one pathogenic variant, c.1798C > T and two predicted pathogenic variants, c.547-27A > G and c.539_543delACTTC. To date, the c.539_543delACTTC variant is unreported but considered as pathogenic. It contains a stop codon in the amino acid sequence with a frameshift mutation due to a defect in 5 bases (Additional file 2). On the other hand, c.1798C > T is a known pathogenic variant [18] and is common in Japanese patients with IOPD [26]. However, the frequency of this allele in this study was 0.9% (2/214), indicating that it is not a hotspot mutation. Human Splicing Finder, which is a tool to predict the effects of mutations on splicing signals or to identify splicing motifs, predicted c.547-27A > G as “potential alteration of splicing.” However, the actual pathogenicity of c.547-27A > G is unknown. Moreover, as the parents of the patient had not been sequenced for the *GAA* gene, which variant was in which allele remains unclear. However, we predicted that the patient has compound heterozygosity for the c.539_543delACTTC and c.1798C > T variants.

Of the seven individuals diagnosed with potential LOPD in this study, six had c. 752C > T + c.761C > T (five heterozygous and two homozygous). c.752C > T + c.761C > T is one of the most frequently detected variants in NBS (allele frequency: 14.5% 31/214). In Taiwan, five of the 13 individuals with potential LOPD were homozygous or heterozygous [27]. The pathogenicity of this variant remains controversial. Chien et al. [28] reported a female patient with c.752C > T + c.761C > T, harboring a potentially pathogenic variant (c.1958C > A) with compound heterozygozity. She presented with proximal muscle weakness without elevated blood CK and urinary glucose tetrasaccharide (Glc4) levels. Individuals with this variant require long-term follow-up to determine variant pathogenicity. Notably, the pathogenic variant c.-32-13T > G, which is frequently detected in patients with LOPD in Europe and the United States [29], was not detected in our screening program.

The total recall rate in this program was 0.1%, which was approximately five times higher than that of the United States [19, 21]. We attribute the high false-positive rate to the high frequency of the pseudodeficiency variant c.[1726G > A; 2065G > A] in East Asia. Our results demonstrated that most of the 107 newborns who underwent genetic analysis had homozygous or heterozygous pseudodeficiency variants (Table 2). A false-positive result constitutes an economic burden and causes subsequent psychological distress on patients and their families. To reduce the false-positive rate, it is necessary to distinguish between pseudodeficient subjects and Pompe disease patients at the time of screening before recall. Liao et al. reported that measuring enzyme activity using tandem mass spectrometry can reduce the incorrect inclusion of individuals with pseudodeficiency among false-positive results [30]. On the other hand, in Illinois, where NBS for Pompe disease was performed by measuring enzyme activity using tandem mass spectrometry, 39 of the 395 individuals who presented with positive screening results were pseudodeficient, and all of them were of Asian descent [20]. Therefore, it may be difficult to distinguish pseudodeficiency only by measuring enzyme activity using tandem mass spectrometry. Additional *GAA* sequencing analysis by next-generation sequencing (NGS) using DBS prior to recall may be effective in reducing the false-positive rate [31]. Furthermore, some studies propose using microRNA (miR-133a) as a biomarker for Pompe disease [32, 33] as it can distinguish patients with IOPD from those with LOPD, indicating its value as a tool for diagnosis and the monitoring of therapeutic effects [33].

This study has some limitations. First, as NBS was conducted in limited areas only, which constitutes approximately 5.4% of the entire population, our results do not reflect the frequency of disease-associated variants throughout Japan. To solve this problem, it is necessary to conduct NBS for Pompe disease throughout Japan. Second, the combination of the enzyme assay and DNA analysis still did not definitively diagnose LOPD patients manifesting no clinically-recognized symptoms. Currently, we are conducting follow-up assessments on individuals with potential LOPD and monitoring their blood CK levels and muscle symptoms to determine if they have LOPD. Additionally, the urinary Glc4 level may be a useful biomarker for Pompe disease [34, 35]. Thus, urinary Glc4 measurements may be considered in future screenings for LOPD.

## Conclusions

We report the current results of NBS for Pompe disease in Japan among 296,759 newborns and identified one patient with IOPD and seven individuals with potential LOPD. The frequency of IOPD in Japan was similar to that in the United States, where the Recommended Uniform Screening Panel (RUSP) suggests NBS for Pompe disease; this indicates that newborn screening for Pompe disease may also be useful in Japan. The IOPD patient immediately underwent ERT within a month after birth before presenting exacerbated manifestations, whereas the seven individuals with potential LOPD underwent long-term follow-up and showed no manifestations. Long-term follow-up from the neonatal period may allow treatment with ERT to begin before irreversible symptoms progress and may, therefore, improve the quality of life of patients. In the future, inter-racial marriage and migration may increase the frequency of Pompe disease. Therefore, NBS for Pompe disease should be extended over a wider region in Japan.

## Materials and methods

### Study population and sample collection

The study population consisted of 297,387 newborns from Kumamoto and Fukuoka prefectures between April 2013 and October 2020. Dried-blood spot (DBS) samples were prepared in each maternity clinic or obstetric department using a heel prick procedure 4–6 days after birth for newborn mass screening across all municipalities. After blotting with blood spots (Toyo Roshi Kaisha, Ltd., Tokyo, Japan), the filter paper was dried for at least 4 h at room temperature (i.e., 20 ± 5 °C), and the samples were sent to the Newborn Screening Center at KM Biologics Co., Ltd. (Kumamoto, Japan), where publicly-funded newborn mass screening was conducted, within 1 week of collection. After analysis, the DBSs after testing were transferred to Kumamoto University to assay GAA activity.

### NBS program for Pompe disease

Our proposed NBS for Pompe disease utilizing GAA assays on DBSs involves three steps (Fig. 1). First, DBS samples whose GAA activity was under the cutoff value (< 6.5 pmol h^−1^ disk^−1^ in Method I; < 3.5 pmol h^−1^ disk^−1^ in Methods II, III) were recalled, and DBSs were prepared again for a second GAA assay. Second, newborns whose GAA activity was under the cutoff value were referred to the hospital within two months for clinical examination, and physical and biochemical assays were performed to confirm the symptomatic signs of IOPD. The new DBS samples were subjected to hemoglobin precipitation using the Ba/Zn method to considerably improve the 4MU fluorescence intensity and a third GAA assay was also performed. Finally, *GAA* gene sequencing was performed in newborns whose GAA activity was under the cutoff value following the third GAA assay to confirm the presence of *GAA* gene variants. Additionally, the GAA activity of the fibroblasts of the newborns was measured between April 2013 and November 2016 [12]. The results of the first GAA assay was acquired 1–2 weeks after birth; the second GAA assay, within 4 weeks after birth; clinical examination, within 2 months after birth; *GAA* gene analysis and final diagnosis, up to 6 months after birth.

### GAA assay

#### Method I

GAA assays on DBSs and fibroblasts were performed as previously described [12]. Briefly, one disk (3.2 mm in diameter) was punched from DBS cards and placed into a well of a 96-well clear microwell plate (Corning, NY, USA) with 100 μL of 0.8 mM citrate in 24 mM potassium phosphate buffer (pH 6.0) containing 0.1% Triton X-100 for 1 h at room temperature with gentle mixing. In a 96-well black microwell-plate (PerkinElmer, Waltham, MA, USA), a 20 μL aliquot of the extract was then added to 40 μL of 2.0 mM 4MU-αGlc in 0.12 M citrate/0.15 M potassium phosphate buffer (pH 4.0) containing 4.5 μM acarbose, incubated at 37 °C for 24 h, and quenched by adding 190 μL of 0.2 M glycine/NaOH buffer (pH 10.7) containing 0.1% Triton X-100 and 0.2% SDS to measure fluorescence intensity.

For the Ba/Zn method, a 3.2-mm diameter disk was placed in a 1.5 mL reaction tube with 60 μL of 0.12 M citrate/0.15 M potassium phosphate buffer (pH 4.0) containing 2.0 mM 4MU-αGlc and 3.0 μM acarbose and gently mixed for 10 min at room temperature. The reaction mixture was incubated at 37 °C for 24 h, added with 30 μL of 0.15 M barium hydroxide, vortexed, and further incubated at room temperature for 5 min. Then, 30 μL of 0.15 M zinc sulfate was added, and the tubes were mixed by vortexing, incubated for 10 min at room temperature, and centrifuged for 5 min at 12,000 rpm and 5 °C. Finally, 90 μL of the supernatant was transferred to a 96-well black microwell plate, and 160 μL of 0.4 M glycine/NaOH buffer (pH 10.7) containing 0.1% Triton X-100 was added to measure fluorescence intensity. Stock solutions of 0, 6.25, 12.5, 25, 50, and 100 μM 4-methylumbelliferone (4MU) in 20 mM sodium phosphate buffer (pH 7.0) were used for standardization of the liberated 4MU concentration. Enzyme activity was expressed as pmol of 4MU released per hour per disk (pmol h^−1^ disk^−1^). Each assay was performed in duplicate.

#### Method II

Method II for multiple assays was developed in collaboration with KM Biologics Co., Ltd. (see details at JP6360848B) and implemented in December 2016. Briefly, a single 3.2-mm diameter disk punched from DBSs was incubated in the well of a 96-well clear microwell plate (Corning, NY, USA) with 100 μL of 5 mM MgCl_2_, 0.5 mM dithiothreitol, 0.05% NaN_3_, and 0.1% Triton X-100 in 0.8 mM citrate/24 mM potassium phosphate buffer (pH 6.0) for 1 h at room temperature with gentle mixing. A 20-μL aliquot of the extract was added to 40 μL of 2.0 mM 4MU-αGlc in 0.12 M citrate/0.15 M potassium phosphate buffer (pH 4.0) containing 4.5 μM acarbose in a 96-well black microwell-plate (PerkinElmer, Waltham, MA, USA). The reaction mixture was incubated at 37 °C for 4 h and quenched by adding 200 μL of 0.3 M glycine/NaOH buffer (pH 10.6) containing 10 mM EDTA to measure fluorescence intensity.

#### Method III

Method III for more high-throughput assays was also developed in collaboration with KM Biologics Co., Ltd. (see details at P2017-245523) and implemented in February 2019. A single 3.2-mm diameter disk punched from DBSs was incubated in the wells of a 96-well clear microwell plate (Corning, NY, USA) with 200 μL of 38 mM KCl, 5 mM MgCl_2_, 0.05% NaN_3_, and 0.1% Triton X-100 in 5 mM sodium acetate buffer (pH 5.2) for 1 h at room temperature with gentle mixing. A 20-μL aliquot of the extract was added to 40 μL of 2.0 mM 4MU-αGlc in 0.12 M citrate/0.15 M potassium phosphate buffer (pH 4.0) containing 4.5 μM acarbose in a 96-well black microwell-plate (PerkinElmer, Waltham, MA, USA). The reaction mixture was incubated at 38 °C for 3 h, and the reaction was stopped by adding 200 μL of 0.3 M glycine/NaOH buffer (pH 10.6) containing 10 mM EDTA to measure fluorescence intensity.

### GAA assay of fibroblasts

GAA assays of fibroblasts were conducted between April 2013 and November 2016. Briefly, fibroblasts were collected from a skin biopsy and cultured under standard conditions in Dulbecco’s modified Eagle’s medium with 10% fetal calf serum and antibiotics (50 kU/L penicillin and 50 mg/L streptomycin). After growing to 100% confluence, fibroblasts were harvested and washed with phosphate-buffered saline. The cell pellet was stored at -80 °C until use. Fibroblasts (2 to 4 × 10^6^ cells) were homogenized in 150 μL of water using sonication on ice, and 10 μL of the cell homogenate was added to 40 μL of the substrate solution containing 2.0 mM 4MU-αGlc in 0.12 M citrate in 0.15 M potassium phosphate buffer (pH 4.0) containing 3.75 μM acarbose (final concentration: 3.0 μM) in a well of a 96-well black microwell-plate. The reaction mixture was incubated at 37 °C for 1 h, and the reaction was stopped by the addition of 200 μL of 0.2 M glycine/NaOH buffer (pH 10.7) containing 0.1% Triton X-100 to measure fluorescence intensity, with blank correction. A stock solution of 250 μM 4MU in 20 mM sodium phosphate buffer (pH 7.0) was used for calibration of the liberated 4MU concentration. Enzyme activity was expressed as nanomoles of 4MU released per hour per milligram of cellular protein (nmol h^−1^ mg^−1^ protein). Each assay was performed in duplicate.

### Chemicals and reagents

4MU-αGlc and 4MU were obtained from Sigma-Aldrich Co. LLC (Tokyo, Japan). Acarbose, barium hydroxide, citric acid monohydrate, dipotassium hydrogenphosphate, disodium hydrogenphosphate 12-water, dithiothreitol, magnesium chloride hexahydrate, sodium azide, sodium dodecyl sulfate, sodium hydrate, Triton X-100, and zinc sulfate were obtained from FUJIFILM Wako Pure Chemical Corporation (Osaka, Japan). Glycine and potassium chloride were obtained from Nacalai Tesque, Inc. (Kyoto, Japan). EDTA was obtained from Dojindo Laboratories (Kumamoto, Japan).

### *GAA* sequencing using NGS

*GAA* sequencing using NGS was conducted as described previously [36]. Briefly, genomic DNA was extracted from total blood using a Gentra Puregene Blood Kit (Qiagen, Hilden, Germany) and stored at − 80 °C until use. The 22-kbp region, including the *GAA*, was amplified by dividing the genomic DNA into three fragments using long-range polymerase chain reaction (Additional file 2: Fig. S2) with DNA polymerase (KOD FX; Toyobo, Osaka, Japan) on a Veriti Thermal Cycler (Applied Biosystems, Foster City, CA, USA). PCR products were purified using an Agencourt AMP XP PCR Purification Kit (Beckman Coulter, Brea, CA, USA) and quantified with a Qubit dsDNA HS Assay Kit (Life Technologies, Carlsbad, CA, USA) using a Qubit 2.0 Fluorometer (Life Technologies). Simultaneous fragmentation of PCR products and adaptor ligation were performed using a Nextera XT Kit (Illumina, San Diego, CA, USA). Indexed DNA was purified using an Agencourt AMP XP PCR Purification Kit (Beckman Coulter). Each library was validated using High Sensitivity D1000 ScreenTape (Agilent Technologies, Santa Clara, CA, USA) with an Agilent 2200 TapeStation and quantified using a Qubit dsDNA HS Assay Kit with a Qubit 2.0 Fluorometer to allow for library normalization. Sequencing was performed with a MiSeq Reagent Kit v3 on MiSeq sequencer (Illumina) using the “paired-end” sequencing run method. Data were aligned to target sequences on the human reference genome sequence using the MiSeq Reporter software (Illumina). Sequence data analysis, mapping, and variant calling were streamlined using MiSeq Reporter v2 (Illumina). Briefly, reads were aligned to the reference sequence from 80,101,882 to 80,123,207 of the genome sequence of chromosome 17 (NC_000017.11) using bwa-0.6.1. Single-nucleotide polymorphism and insertion/deletion identifications were performed using the Genome Analysis Toolkit (GATK v1.6; Broad Institute, Cambridge, MA, USA), and visualization was performed using IGV_2.3.40 (Broad Institute).

### *GAA* resequencing using the Sanger method

Variants detected in the *GAA* gene using NGS were re-sequenced using the Sanger method [13]. Briefly, a region including the variant was amplified using PCR with an appropriate set of primers. PCR products were sequenced on an ABI3500xl auto sequencer (Applied Biosystems) and analyzed using Sequencher 5.0 (Gene Codes Corporation, Foster City, CA, USA).

### Mutation analysis of the variants

The mRNA reference sequence (RefSeq) NM_000152.4 was used, whereby the “A” nucleotide of the ATG codon at nucleotide position 398 of the RefSeq constituted + 1 numbering of the cDNA sequence. The ATG codon was also represented as + 1 for amino acid numbering as set forth by the GAA preprotein sequence NP_000143.2. Mutation nomenclature followed the guidelines established by the Human Genome Variation Society (http://varnomen.hgvs.org/). The Pompe disease *GAA* variant database [13] (http://www.pompevariantdatabase.nl; Accessed March 3, 2021) and the ClinVar Miner [14] (https://clinvarminer.genetics.utah.edu/; Accessed March 3, 2021) were to classify each variant. PolyPhen-2 [37] (http://genetics.bwh.harvard.edu/pph2; Accessed March 3, 2021) was used to predict the potential effect of an amino acid alteration on the function of GAA. The online bioinformatics tool, Human Splicing Finder [16] (http://www.umd.be/HSF3/; Accessed March 3, 2021) was used to estimate the effects of mutations on splicing signals.

## Supplementary Information

**Additional file 1: Fig. S1**. Histograms of acid α-glucosidase (GAA) activity in the newborns. Histograms of GAA activity are shown for (a) Method II (*N* = 113,642) and (b) Method III (*N* = 82,208) in the newborns. Dashed line indicates the cutoff level.

**Additional file 2: Fig. S2**. Frameshift variant, c.539_543delACTTC. It contains a stop codon in the amino acid sequence with a frameshift mutation due to a defect in 5 bases.

**Additional file 3**:** Fig. S3**. Long-range PCR of *GAA* gene. The broad black bars and narrow black bars indicate exons and introns, respectively; the horizontal black bar and blue bars indicate the *GAA* gene, and pairs of primers, respectively. The ATG codon is represented as + 1 for amino acid numbering as per the GAA preprotein sequence NP_000143.2

## Data Availability

The datasets used and/or analysed during the current study are available from the corresponding author on reasonable request.

## Abbreviations

GAA: Acid α-glucosidase
NBS: Newborn screening
IOPD: Infantile-onset Pompe disease
LOPD: Late-onset Pompe disease
ERT: Enzyme replacement therapy
DBSs: Dried blood spots
4MU-αGlc: 4-Methylumbelliferyl α-d-glucopyranoside
IQR: Interquartile range
CK: Creatine kinase
Glc4: Glucose tetrasaccharide
NGS: Next-generation sequencing
4MU: 4-Methylumbelliferone
RefSeq: Reference sequence
