# Supplementary material for: Current status of newborn screening for Pompe disease in Japan
Source: Orphanet J Rare Dis. 2021 Dec 18;16:516. doi: 10.1186/s13023-021-02146-z (PMC8684119; doi:10.1186/s13023-021-02146-z)
Supplement: Supplementary file 1 — Additional file 1: Fig. S1. Histograms of acid α-glucosidase (GAA) activity in the newborns. Histograms of GAA activity are shown for (a) Method II (N = 113,642) and (b) Method III (N = 82,208) in the newborns. Dashed line indicates the cutoff level. [file 13023_2021_2146_MOESM1_ESM.pptx]

## Slide 1
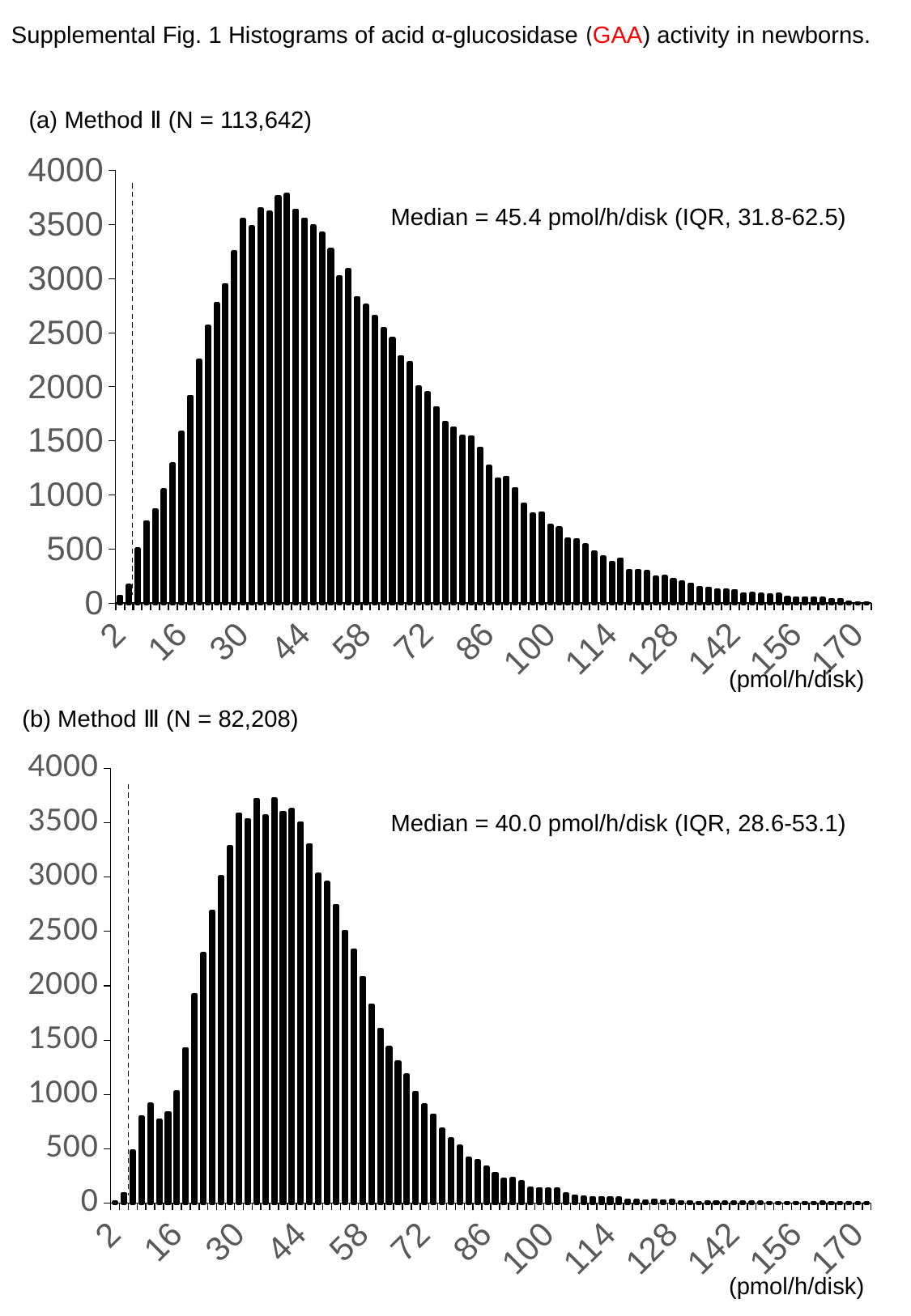

Supplemental Fig. 1 Histograms of acid α-glucosidase (GAA) activity in newborns.
 (a) Method Ⅱ (N = 113,642)
### Chart
| Category | |
|---|---|
| 2 | 61.0 |
| 4 | 163.0 |
| 6 | 497.0 |
| 8 | 749.0 |
| 10 | 859.0 |
| 12 | 1044.0 |
| 14 | 1283.0 |
| 16 | 1575.0 |
| 18 | 1909.0 |
| 20 | 2243.0 |
| 22 | 2556.0 |
| 24 | 2766.0 |
| 26 | 2941.0 |
| 28 | 3244.0 |
| 30 | 3546.0 |
| 32 | 3475.0 |
| 34 | 3640.0 |
| 36 | 3610.0 |
| 38 | 3754.0 |
| 40 | 3778.0 |
| 42 | 3631.0 |
| 44 | 3548.0 |
| 46 | 3482.0 |
| 48 | 3417.0 |
| 50 | 3267.0 |
| 52 | 3012.0 |
| 54 | 3080.0 |
| 56 | 2822.0 |
| 58 | 2751.0 |
| 60 | 2646.0 |
| 62 | 2536.0 |
| 64 | 2444.0 |
| 66 | 2275.0 |
| 68 | 2221.0 |
| 70 | 1996.0 |
| 72 | 1941.0 |
| 74 | 1805.0 |
| 76 | 1668.0 |
| 78 | 1617.0 |
| 80 | 1538.0 |
| 82 | 1535.0 |
| 84 | 1430.0 |
| 86 | 1263.0 |
| 88 | 1146.0 |
| 90 | 1162.0 |
| 92 | 1057.0 |
| 94 | 913.0 |
| 96 | 821.0 |
| 98 | 831.0 |
| 100 | 718.0 |
| 102 | 694.0 |
| 104 | 591.0 |
| 106 | 583.0 |
| 108 | 536.0 |
| 110 | 472.0 |
| 112 | 423.0 |
| 114 | 374.0 |
| 116 | 406.0 |
| 118 | 297.0 |
| 120 | 300.0 |
| 122 | 292.0 |
| 124 | 236.0 |
| 126 | 244.0 |
| 128 | 216.0 |
| 130 | 192.0 |
| 132 | 174.0 |
| 134 | 142.0 |
| 136 | 137.0 |
| 138 | 120.0 |
| 140 | 119.0 |
| 142 | 113.0 |
| 144 | 84.0 |
| 146 | 88.0 |
| 148 | 80.0 |
| 150 | 73.0 |
| 152 | 79.0 |
| 154 | 51.0 |
| 156 | 47.0 |
| 158 | 47.0 |
| 160 | 47.0 |
| 162 | 42.0 |
| 164 | 32.0 |
| 166 | 33.0 |
| 168 | 8.0 |
| 170 | 0.0 |
| 172 | 2.0 |Median = 45.4 pmol/h/disk (IQR, 31.8-62.5)
(pmol/h/disk)
(b) Method Ⅲ (N = 82,208)
### Chart
| Category | |
|---|---|
| 2 | 9.0 |
| 4 | 86.0 |
| 6 | 477.0 |
| 8 | 790.0 |
| 10 | 906.0 |
| 12 | 762.0 |
| 14 | 829.0 |
| 16 | 1022.0 |
| 18 | 1419.0 |
| 20 | 1915.0 |
| 22 | 2293.0 |
| 24 | 2683.0 |
| 26 | 3001.0 |
| 28 | 3278.0 |
| 30 | 3579.0 |
| 32 | 3526.0 |
| 34 | 3712.0 |
| 36 | 3559.0 |
| 38 | 3717.0 |
| 40 | 3594.0 |
| 42 | 3618.0 |
| 44 | 3496.0 |
| 46 | 3295.0 |
| 48 | 3025.0 |
| 50 | 2948.0 |
| 52 | 2738.0 |
| 54 | 2499.0 |
| 56 | 2327.0 |
| 58 | 2073.0 |
| 60 | 1822.0 |
| 62 | 1597.0 |
| 64 | 1429.0 |
| 66 | 1298.0 |
| 68 | 1176.0 |
| 70 | 1017.0 |
| 72 | 901.0 |
| 74 | 808.0 |
| 76 | 680.0 |
| 78 | 587.0 |
| 80 | 519.0 |
| 82 | 412.0 |
| 84 | 385.0 |
| 86 | 329.0 |
| 88 | 271.0 |
| 90 | 218.0 |
| 92 | 225.0 |
| 94 | 194.0 |
| 96 | 135.0 |
| 98 | 131.0 |
| 100 | 127.0 |
| 102 | 128.0 |
| 104 | 82.0 |
| 106 | 63.0 |
| 108 | 52.0 |
| 110 | 49.0 |
| 112 | 49.0 |
| 114 | 46.0 |
| 116 | 43.0 |
| 118 | 25.0 |
| 120 | 24.0 |
| 122 | 17.0 |
| 124 | 26.0 |
| 126 | 18.0 |
| 128 | 22.0 |
| 130 | 12.0 |
| 132 | 11.0 |
| 134 | 3.0 |
| 136 | 9.0 |
| 138 | 9.0 |
| 140 | 5.0 |
| 142 | 5.0 |
| 144 | 6.0 |
| 146 | 8.0 |
| 148 | 5.0 |
| 150 | 4.0 |
| 152 | 4.0 |
| 154 | 3.0 |
| 156 | 3.0 |
| 158 | 3.0 |
| 160 | 2.0 |
| 162 | 5.0 |
| 164 | 2.0 |
| 166 | 1.0 |
| 168 | 3.0 |
| 170 | 0.0 |
| 172 | 1.0 |Median = 40.0 pmol/h/disk (IQR, 28.6-53.1)
(pmol/h/disk)
