# Supplementary material for: Current status of newborn screening for Pompe disease in Japan
Source: Orphanet J Rare Dis. 2021 Dec 18;16:516. doi: 10.1186/s13023-021-02146-z (PMC8684119; doi:10.1186/s13023-021-02146-z)
Supplement: Supplementary file 2 — Additional file 2: Fig. S2. Frameshift variant, c.539_543delACTTC. It contains a stop codon in the amino acid sequence with a frameshift mutation due to a defect in 5 bases. [file 13023_2021_2146_MOESM2_ESM.pptx]

## Slide 1
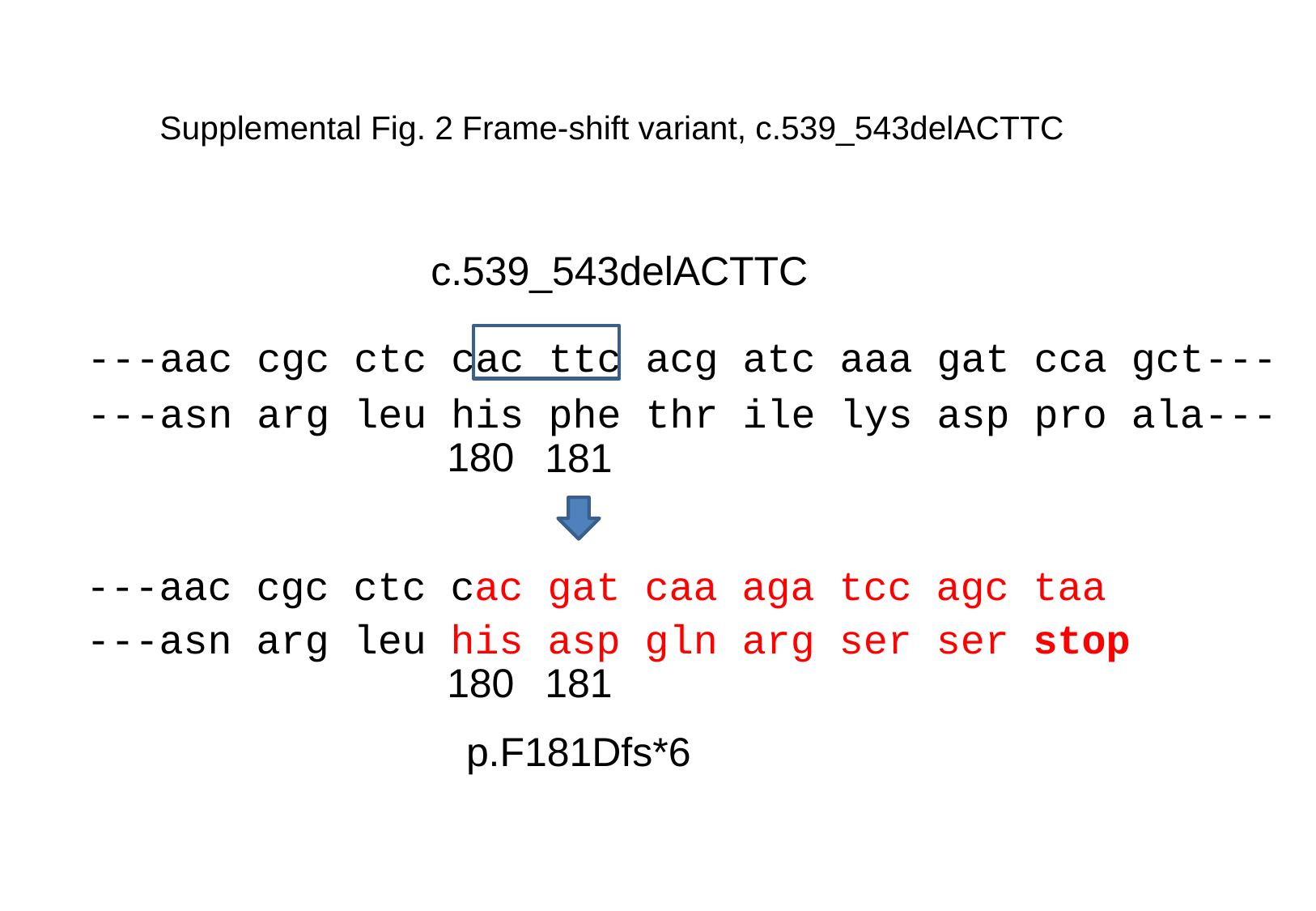

Supplemental Fig. 2 Frame-shift variant, c.539_543delACTTC
c.539_543delACTTC
---aac cgc ctc cac ttc acg atc aaa gat cca gct---
---asn arg leu his phe thr ile lys asp pro ala---
180
181
---aac cgc ctc cac gat caa aga tcc agc taa
---asn arg leu his asp gln arg ser ser stop
181
180
p.F181Dfs*6
