# Supplementary material for: Current status of newborn screening for Pompe disease in Japan
Source: Orphanet J Rare Dis. 2021 Dec 18;16:516. doi: 10.1186/s13023-021-02146-z (PMC8684119; doi:10.1186/s13023-021-02146-z)
Supplement: Supplementary file 3 — Additional file 3: Fig. S3. Long-range PCR of GAA gene. The broad black bars and narrow black bars indicate exons and introns, respectively; the horizontal black bar and blue bars indicate the GAA gene, and pairs of primers, respectively. The ATG codon is represented as + 1 for amino acid numbering as per the GAA preprotein sequence NP_000143.2 [file 13023_2021_2146_MOESM3_ESM.pptx]

## Slide 1
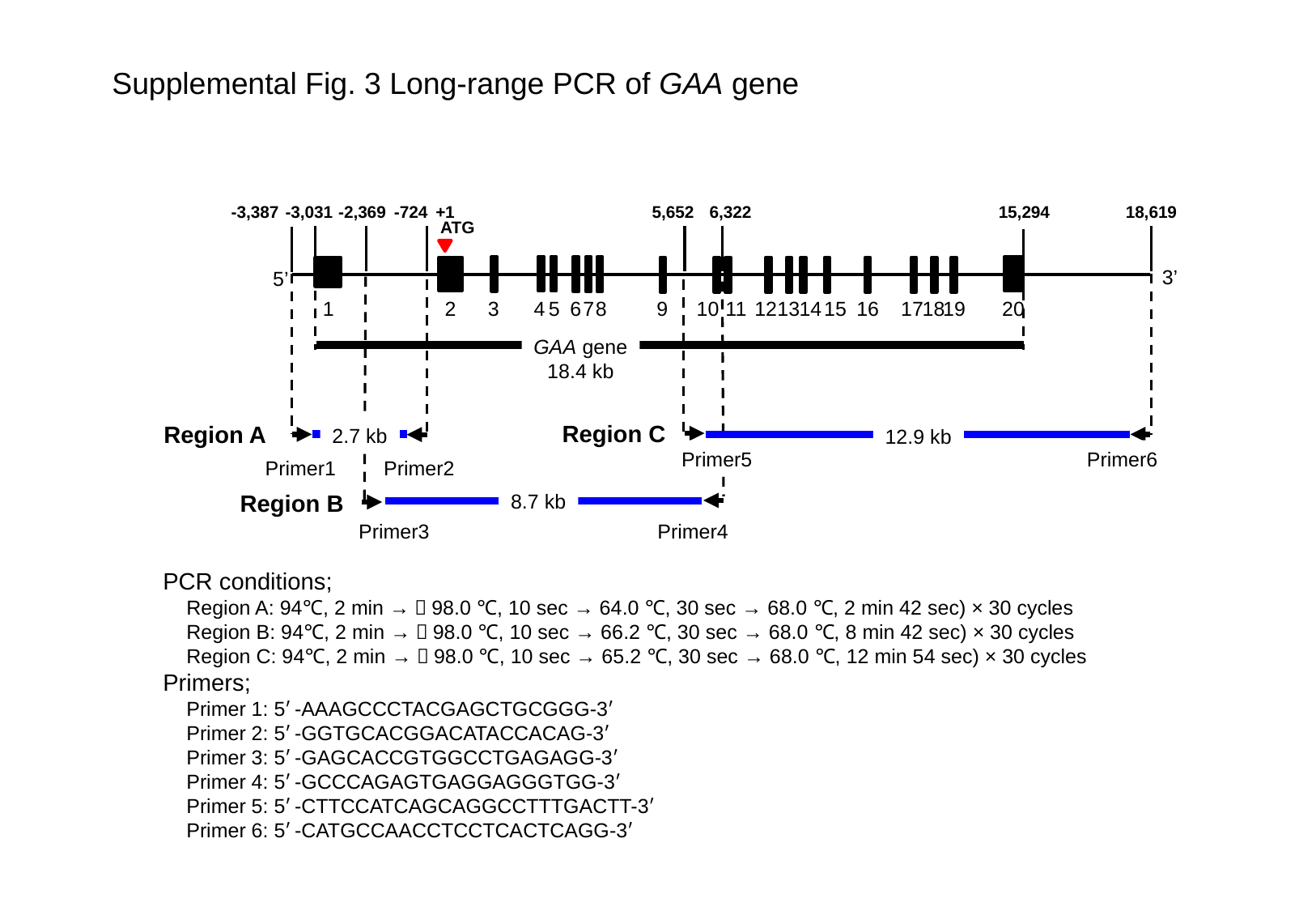

Supplemental Fig. 3 Long-range PCR of GAA gene
-2,369
-724
+1
-3,387
-3,031
5,652
6,322
15,294
18,619
ATG
3’
5’
1
2
3
4
5
6
7
8
9
10
11
12
13
14
15
16
17
18
19
20
GAA gene
18.4 kb
Region C
Region A
2.7 kb
12.9 kb
Primer5
Primer6
Primer1
Primer2
Region B
8.7 kb
Primer3
Primer4
PCR conditions;
Region A: 94℃, 2 min →（98.0 ℃, 10 sec → 64.0 ℃, 30 sec → 68.0 ℃, 2 min 42 sec) × 30 cycles
Region B: 94℃, 2 min →（98.0 ℃, 10 sec → 66.2 ℃, 30 sec → 68.0 ℃, 8 min 42 sec) × 30 cycles
Region C: 94℃, 2 min →（98.0 ℃, 10 sec → 65.2 ℃, 30 sec → 68.0 ℃, 12 min 54 sec) × 30 cycles
Primers;
Primer 1: 5ʹ -AAAGCCCTACGAGCTGCGGG-3ʹ
Primer 2: 5ʹ -GGTGCACGGACATACCACAG-3ʹ
Primer 3: 5ʹ -GAGCACCGTGGCCTGAGAGG-3ʹ
Primer 4: 5ʹ -GCCCAGAGTGAGGAGGGTGG-3ʹ
Primer 5: 5ʹ -CTTCCATCAGCAGGCCTTTGACTT-3ʹ
Primer 6: 5ʹ -CATGCCAACCTCCTCACTCAGG-3ʹ
